# Supraclavicular Artery Flap versus Skin Graft: Which Is a Better Reconstructive Tool for Managing Post-Burn Contractures in the Neck

**DOI:** 10.29252/wjps.10.1.15

**Published:** 2021-01

**Authors:** Muhammad Saaiq

**Affiliations:** 1 Department of Plastic Surgery and Burns, National Institute of Rehabilitation Medicine (NIRM), Islamabad, Pakistan.

**Keywords:** Burn, Contracture, Supraclavicular artery flap, Skin graft

## Abstract

**BACKGROUND:**

Burn in developing countries still has high burden of inadequately managed severe burns. This study compared supraclavicular artery flap and skin graft in managing neck post-burn contractures.

**METHODS:**

In National Institute of Rehabilitation Medicine and Pakistan Institute of Medical Sciences, Islamabad, Pakistan, 30 patients with neck post-burn contractures were enrolled. Half of patients randomly underwent supraclavicular artery flap and half received skin graft. The outcome measures including initial improvement in neck extension, patient’s satisfaction with color-texture-match and recurrent contracture formation rate were assessed.

**RESULTS:**

Among patients, 80% were female and 20% were male. Preoperatively, each group had post-burn contractures of grade II among 26.66% of patients, grade III among 60% and grade III among 13.3%. Postoperatively after three months in the two groups, 86.66% improved to grade I and 13.3% improved to grade II. Patient’s satisfaction with color-texture was 84.66% in supraclavicular artery flap group, whereas it was 42.66% for skin graft group. Complications were hypertrophic scar at donor site (13%) and flap tip necrosis (6.66%) in supraclavicular artery flap group. In skin graft group, partial skin graft loss was noticed among 33% of patients and delayed healing of donor site among 20%. The recurrent contracture formation rate at one year was 73.33% in skin graft group, whereas there was no case of recurrent contracture in supraclavicular artery flap group.

**CONCLUSION:**

Supraclavicular artery flap was superior to skin graft in managing post-burn neck contractures. It provided better color-texture match and was associated with no recurrence of contracture formation.

## INTRODUCTION

Burn care has considerable advances in the developed world, however, in the developing nations, it still continues to have high burden of severe burns. There is still lack of ideal facilities for managing acute burns and the sequelae of burns. So post-burn contractures (PBCs) are often severe and neglected for a long period of time.^1^^-^^3^

The anterior of neck region represents one major anatomic territory of the body which is frequently affected in major burns.^1^

When the deep burn of this region is allowed to heal by secondary intention with dressings for weeks to months, severe scarring ensues that invariably leads to the development of flexion contracture. There are additional factors that predispose to the formation of PBCs in the neck including the thin and soft skin of the neck, the superficially located platysma muscle and the relative ease of flexion posture at the neck. The neck post-burn contractures have serious repercussions for the sufferer. It is not only functionally incapacitating, it is cosmetically disfiguring too. It restricts the range of neck motion and also impairs the acts of mastication, deglutition and social interactions. The lips, chin and cheeks are pulled downward. Among children of growing age group, the growth of both the mandible and cervical spine is negatively affected too. Overall the sufferer has a distorted self-image.^1^^,^^4^^-^^6^

The neck post-burn contractures pose unique management challenges to the plastic surgeon. The guiding principle is to ensure complete release of the contracture and restore form and function to this critically visible part of the body. The various reconstructive options available ranged from skin grafts to local and free flaps. Each of these reconstructive tools has its own attended advantages, disadvantages and limitations.^2^^,^^5^^,^^7^-10 So this study compared the outcome of supraclavicular artery flap versus skin graft in managing neck post-burn contractures. The outcome measures scrutinized included improvement in neck extension at three months, patient’s satisfaction with color-texture match and the rate of recurrent contracture formation at one year follow-up.

## MATERIALS AND METHODS

This comparative study was conducted at the National Institute of Rehabilitation Medicine (NIRM) and Pakistan Institute of Medical Sciences (PIMS), Islamabad, Pakistan from January 2014 to December 2018. Informed consent was taken from the patients. The prospective comparative study proceeded in accordance with the Helsinki’s Declaration-2013 revision. We ensured anonymity of the participants. The study included all neck post-burn contractures patients (n=30) who were managed during the study period. 

Half of the patients were assigned to supraclavicular artery flap group and half to the skin graft group. Simple random sampling was done with computer generated random number table. The two groups were matched for the demographic features and contracture characteristics. The following “neck extension-deficit grading system”11 was employed for assessing the grade of post-burn contractures and improvement in extension following surgery.

Grade 1 was considered normal extension of >110°, Grade 2 as neck extension beyond the horizontal plane, parallel to the ground (i.e. 95°-110°), Grade 3 defined as neck extension and vision limited to the horizontal plane only (i.e. 85°-95°) and Grade 4 was regarded mentosternal synechia/visual range below the horizontal plane (i.e. neck extension <85°). All patients underwent the surgery under general anesthesia. In supraclavicular artery flap group, where supraclavicular artery flap was employed, the patient was positioned supine on the operating table at the time of surgery. 

The neck and shoulder were prepped and draped. The triangle where flap pedicle originated was marked. It was bounded medially by the posterior border of the sternocleidomastoid, posteriorly by the external jugular vein and inferiorly by the clavicle. The flap was marked on the patient’s shoulder after taking template of the defect resulting from release of the neck post-burn contractures. The skin, subcutaneous tissue and fascia were incised over the deltoid muscle and flap elevation performed in the sub-fascial plane in a distalo-proximal direction. 

The dissection was continued as far as the supraclavicular fossa, where the supraclavicular artery was identified under the posterior belly of omohyoid muscle. The supraclavicular artery was confirmed with trans-illumination in the middle 3rd of the flap. The spinal accessory nerve, which crossed the deep portion of the posterior triangle of the neck underneath the sternocleidomastoid muscle preserved. Proximally an intact skin border was left over the flap pedicle, while the inferior side was joined to the defect. 

This technique helped to prevent compression and twisting of the flap’s vascular pedicle. The elevated flap was brought onto the defect through 180° rotation. Dimensions of the harvested supraclavicular artery flaps ranged from 16×7 cm (112 cm2) to 25×7 cm (175 cm2). The donor defect was closed primarily among all cases after wide undermining of the edges. In skin graft group, sheets of thick split thickness skin grafts, harvested from the thigh were employed for covering the defects resulting from contracture-release.

In the immediate postoperative period, the patients were advised to lie supine while sleeping, without employing a pillow. Custom-made Watusi neck collar was worn by all patients during day time and continued for 3 months. The skin staples/stitches were removed on 10th postoperative day. The patients were followed-up regularly at 3 monthly intervals. At each follow up, evaluation was performed for skin graft take/flap status, range of neck motion and any complications. Secondary procedures for any complications were undertaken where indicated.

The postoperative improvement in neck extension and patients’ satisfaction were evaluated at three months. For objectively measuring the patients’ satisfaction with the color-texture match of the skin graft versus supraclavicular artery flap, we employed a 10-points response scale. The score ranged from 1 to 10. The average of these responses was calculated for each group and then projected to percentage score out of 100. The rate of recurrent contracture formation was evaluated at one year postoperatively.

The sociodemographic profile of the patients, interventions instituted and outcomes were all recorded. The data were subjected to statistical analysis using SPSS software (version 11, Chicago, IL, USA). Chi Square test was employed to analyse the data and p<0.05 was considered statistically significant.

## RESULTS

Among enrolled 30 patients, 24 (80%) were female and 6 (20%) were male. The age ranged from 16 to 45 years with a mean of 23.7±9.71 years. The post-burn contractures were secondary to flame burns among all patients. Pre-operatively, each group had post-burn contractures of grade II among 4 (26.66%) patients, grade III among 9 (60%) patients and grade III among 2 (13.3%) patients. Post-operatively, improvement in the neck extension at six months was matching for the two groups, with 13 patients (86.66%) improved to grade I and two patients (13.3%) improved to grade II.

**Fig. 1:**
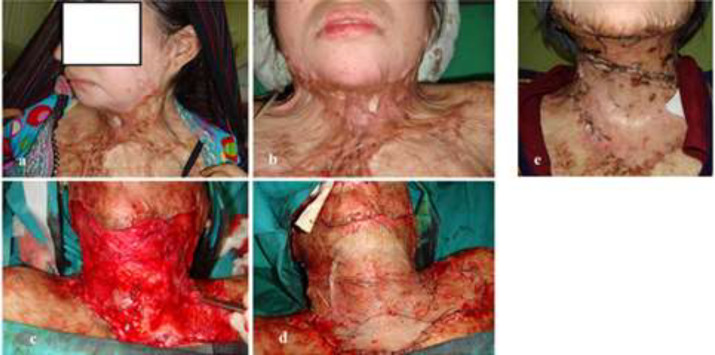
**(a-e):** A young lady with neck post-burn contractures. She sustained flame burns in kitchen, which were treated conservatively at another healthcare facility. The scarred neck precluded the possibility of supraclavicular artery flap in her case. Thick split thickness skin graft sheets were employed to resurface the defect following release of contracture

**Fig. 2:**
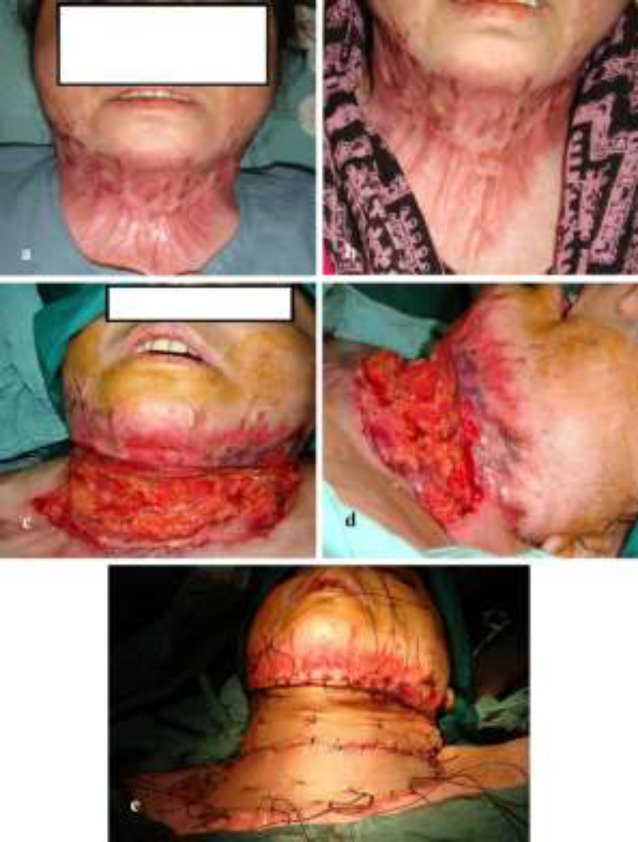
**(a-e):** A young lady with neck post-burn contractures. Sheets of thick split thickness skin graft were used to resurface the defect following release of contracture

**Fig. 3:**
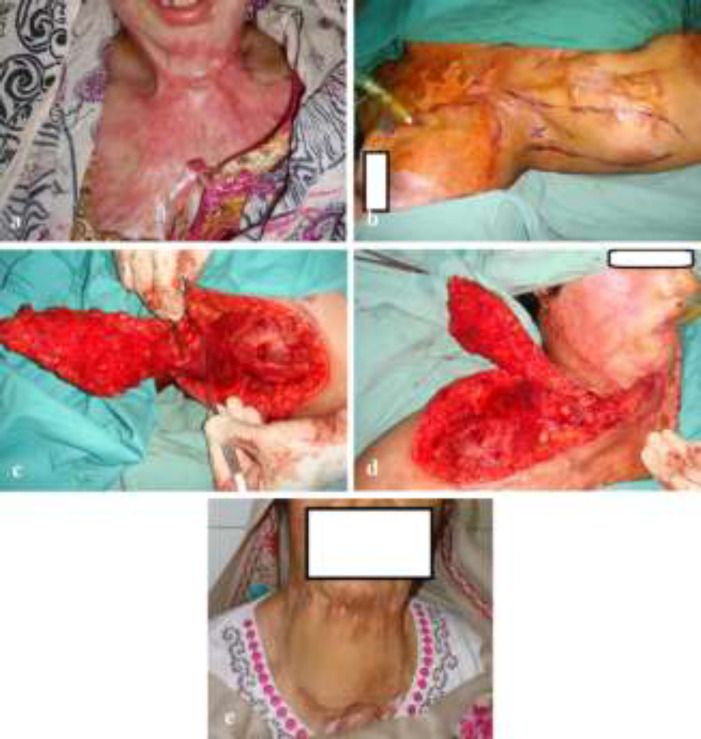
**(a-e):** A young lady developed neck post-burn contractures after sustaining flame burns in kitchen. She was managed conservatively for initial burn injury at another hospital. A right sided supraclavicular artery flap was employed in her case

**Fig. 4:**
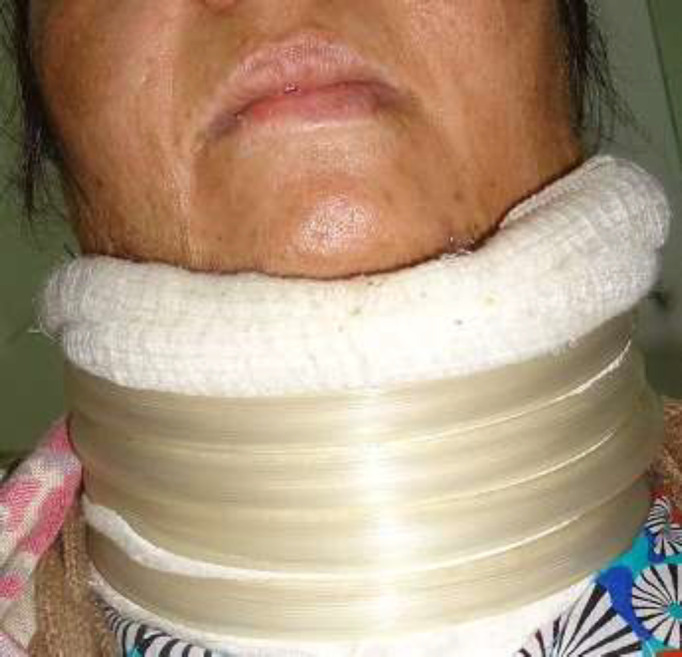
Custom-made Watusi splint employed among all cases during the first three months, postoperatively

Patient’s satisfaction with the color-texture match was 84.66% in supraclavicular artery flap group, whereas it was 42.66% for the skin graft group. Complications encountered included hypertrophic scarring at donor sites (n=2, 13%) and flap tip necrosis (n=1, 6.66%) in supraclavicular artery flap. In skin graft group, we encountered partial skin graft loss among 5 (33%) patients and delayed healing of donor site among 3 (20%) patients. The rate of recurrent contracture formation at one year was 73.33% in skin graft group, whereas there was no case of recurrent contracture formation in supraclavicular artery flap group (Figures 1-4). 

## DISCUSSION

Our study reflected the high burden of neck post-burn contractures in our country. Our finding conforms to published papers from several other developing nations.^2^^,^^9^^,^^10^ In our study, we observed more frequent affliction of females with neck post-burn contractures. Similar predominant affliction of females was also reported by other studies. For instance, Mody et al. from India^2^, Loghmani et al. from Iran^9^ and Ismail et al. from Egypt^10^ have reported more frequent involvement of women with neck post-burn contractures. The exact explanation to this is hitherto not known, however, it may reflect the household practices of women in these societies and hence, their high vulnerability to sustaining flame burns in their primitive style kitchens.^2^^,^^9^^,^^10^

In our study, majority of the patients belonged to the younger stratum of the population. Several published studies have described similar frequent involvement of younger individuals. For instance, Mody et al. from India reported a mean age of 28.2 years (age range of 13-53 years),^2^ Loghmani et al. from Iran reported mean age of 24.62 years (age range of 12-46 years)^9^ whereas, Ismail et al. from Egypt reported mean age of 25.5 years (age range of 48-16 years).10 The socio-economic implications of the neck post-burn contractures were even more serious given the frequent involvement of the economically more productive stratum of the population.^2^^,^^9^^,^^10^

We employed the “neck extension-deficit” grading system for evaluating the intensity of extension-deficit. This system serves to practically assess the function. The grades define the functional impairment and help to compare the improvement after surgery.^11^ We employed the supraclavicular artery flap among half of our patients. This thin, hairless and pliable flap provides an ideal color-texture match to that of the facial skin. 

The flap has come a long way to evolve to its present day status of being a well-established axial pattern flap. Its detailed anatomy is well described in many papers. The vascular anatomy is briefly described herein. The supraclavicular artery is a perforator branch originating from the transverse cervical artery in 93% individuals whereas from the suprascapular artery in the remainder 7% of individuals. The transverse cervical artery branches from the thyrocervical trunk, which in turn originates from the 3rd part of the subclavian artery.^8^^,^^12^^-^^14^

The supraclavicular artery has a diameter of 1-1.5 mm and measures 3-4 cm in length. The artery is located in a triangle outlined by the posterior border of the sternocleidomastoid muscle medially, the external jugular vein posteriorly and the median 3rd of the clavicle inferiorly. The artery arises 3 cm proximal to the clavicle, about 8 cm away from the sternoclavicular junction. The artery is accompanied by two venae commitantes.^8^^,^^12^^-^^14^

In our study, the flap dimensions ranged from 16×7 cm (112 cm2) to 25×7 cm (175 cm2).

Ismail et al. from Egypt employed a mean flap length of 21.7±2.52 cm (range of 16-25 cm), with a mean width of 9.7±1.17 cm (range of 7-11 cm) in their series.10 Loghmani et al. from Iran reported flap length of 18±6 cm and width of 9±3 cm.^9^ Vinh et al. reported flap size of 20±8 cm in length and 10±4 cm in width.12 Kokot et al. reported mean flap dimensions of 6.1 cm (range: 5-9 cm) in width and 21.4 cm (range: 15-28 cm) in length for oncologic defects of the head and neck.^7^ Telang et al. reported safe elevation of supraclavicular artery flap within dimensions of 20×10 cm.15 Use of tissue expansion greatly amplified the total flap area available for flap harvest.^7^^-^^10^^,^^15^

We had one case of venous congestion and distal necrosis of supraclavicular artery flap in our series. Vinh et al. reported distal necrosis in 3 out of their 32 patients. Two of them healed spontaneously, whereas as the third one required coverage.^12^ Ismail et al. reported partial distal necrosis in 7 cases, where 5 had superficial epidermolysis and 2 had full thickness necrosis.^10^ Loghmani et al. in a study with 41 flaps, had 3 cases of distal necrosis.9 Kokot reported 2 (4%) cases with complete flap necrosis.7 Others reported that the flaps lengths of ≥23 cm were strongly correlated with the risk of distal necrosis.^10^^,^^16^

In our study, we employed sheets of thick split thickness skin grafts in cases where the feasibility of supraclavicular artery flap was precluded by scarring of both supraclavicular regions secondary to the initial burn insults. The skin grafts constituted a last option, when other reconstructive tools were not feasible from the patient’s or surgeon’s perspective. Although they were less time consuming and technically less demanding, they were attended by very high rate of recurrent contracture formation. Also, these were cosmetically less acceptable to the patients. Our findings are in line with several other atudies.^17^^-^^20^

## CONCLUSION

The supraclavicular artery flap was shown to be superior to skin graft in managing post-burn neck contractures. It provided better color-texture match and was associated with no recurrence of contracture formation. Initial improvement in neck extension at three months matched for the two groups; however, recurrent contractures developed in the majority of the skin grafted patients at one year follow-up.
